# NGMA-6. Quantitative MGMT Promoter Methylation Index Indicates Non-Linear, Prognostic Effect in Glioblastoma

**DOI:** 10.1093/noajnl/vdab070.021

**Published:** 2021-07-05

**Authors:** David Gibson, Akshay Ravi, Eduardo Rodriguez, Susan Chang, Nancy Bush, Jennie Taylor, Jennifer Clarke, David Solomon, Aaron Scheffler, John Witte, Hideho Okada, Mitchel Berger, Farid Chehab, Nicholas Butowski

**Affiliations:** University of California, San Francisco, San Francisco, CA, USA

## Abstract

**Background:**

Epigenetic inhibition of the O6-methylguanine-DNA-methyltransferase (MGMT) gene has emerged as a clinically relevant prognostic marker in glioblastoma (GBM). Methylation of the MGMT promoter has been shown to increase chemotherapy efficacy. While traditionally reported as a binary marker, recent methodological advancements have led to quantitative methods of measuring methylation, allowing for clearer insights into methylation’s functional relationship with survival.

**Methods:**

A CLIA assay and bisulfite sequencing was utilized to develop a quantitative, 17-point MGMT promoter methylation index derived from the number of methylated CpG sites. Retrospective review of 242 newly diagnosed GBM patients was performed in order to discern how risk for mortality transforms as promoter methylation increases. Non-linearities were captured by fitting splines to Cox proportional hazard models, plotting smoothed residuals, and creating survival plots. Covariates included age, KPS, IDH1 mutation, and extent of resection.

**Results:**

Median follow-up time and progression free survival were 15.9 and 9 months, respectively. 176 subjects experienced death. A one-unit increase in CpG methylation resulted in a 4% reduction in hazard (95% CI 0.93–0.99, P<0.005). Moreover, GBM patients with low levels of methylation (1–6 CpG islands) fared markedly worse (HR=1.62, 95% CI 1.03–2.54, P<0.036) than individuals who were unmethylated (reference group). Subjects with medium levels of methylation (7–12 sites) had the greatest reduction in hazard (HR=0.48, 95% CI 0.29–0.80, P<0.004), followed by individuals in the highest methylation tertile (HR=0.62, 95% CI 0.40–0.97, P<0.035).

**Conclusion:**

The extent of MGMT methylation shares a non-linear relationship with survival, suggesting conformation of the marker’s protective effect. This finding challenges the current understanding of MGMT and underlines the clinical importance of determining its prognostic utility. Potential limitations include censoring, sample size, and extraneous mutations. Future research is warranted to examine whether the location of CpG site methylation contributes to a reduction in mortality hazard.

